# Upregulation of PRRX2 by silencing Marveld3 as a protective mechanism against radiation-induced ferroptosis in skin cells

**DOI:** 10.1186/s10020-024-00958-w

**Published:** 2024-10-21

**Authors:** Jinming Cao, Mengyao Wu, Wei Mo, Min Zhao, Liming Gu, Xi Wang, Bin Zhang, Jianping Cao

**Affiliations:** 1 Department of Nuclear Medicine, The First Affiliated Hospital of Soochow University, Suzhou, China; 2 Department of Oncology, The First Affiliated Hospital of Soochow University, Suzhou, China; 3 School of Radiation Medicine and Protection, Suzhou Medical College of Soochow University, Suzhou, China; 4 State Key Laboratory of Radiation Medicine and Protection, Soochow University, Suzhou, China

**Keywords:** Radiation-induced skin injury, Marveld3, PRRX2, Ferroptosis

## Abstract

**Background:**

Radiation-induced skin injury (RISI) represents a significant complication in patients receiving radiotherapy and individuals exposed to nuclear accidents, characterized by a protracted wound-healing process relative to injuries from other etiologies. Current preventive and management approaches remain inadequate. Consequently, investigating efficacious intervention strategies that target the disease’s progression characteristics holds significant practical importance.

**Methods:**

Small interfering RNA (siRNA) and overexpression plasmid were used to modulate the expression of Marvel domain containing 3 (Marveld3) and paired related homeobox 2 (PRRX2). Protein and mRNA levels were estimated by Western Blot and real-time PCR, respectively. Intracellular levels of Malondialdehyde (MDA), a terminal product of lipid peroxidation, were measured following the manufacturer’s protocol for MDA assay kit. Similarly, intracellular levels of ferrous iron (Fe^2+^) and reactive oxygen species (ROS) were determined using their respective assay kits. Lipid peroxidation status within the cells was evaluated via BODIPY staining. Immunohistochemistry was conducted to ascertain the expression of PRRX2 in skin tissues collected at various time points following irradiation of rats. The H-score method was used to evaluate the percentage of positively stained cells and staining intensity. RNA sequencing, Gene Ontology (GO) analysis, and Kyoto Encyclopedia of Genes and Genomes (KEGG) pathway enrichment analysis were conducted by OE Biotech Company.

**Results:**

In this study, our findings indicated that Marveld3 suppression could effectively inhibit lipid peroxidation levels in irradiated skin cells, concomitantly reducing intracellular Fe^2+^ content. Additionally, the silencing of Marveld3 effectively abrogated the impact of a ferroptosis agonist on cellular viability, resulting in the upregulation of 66 and 178 genes, as well as the downregulation of 188 and 31 genes in irradiated HaCaT and WS1 cells, respectively. Among the differentially expressed genes, the PRRX2 which was found to be involved in the process of ferroptosis, exhibited statistically significant upregulation. And the upregulation of PRRX2 expression may attenuate radiation-induced lipid peroxidation in skin cells, thereby functioning as a potential stress-responsive mechanism to counteract radiation effects.

**Conclusions:**

This study elucidates the role of Marveld3 in radiation-induced ferroptosis in skin cells. Inhibition of Marveld3 led to the upregulation of PRRX2, which subsequently resulted in a reduction of Fe^2+^ and ROS levels, as well as the suppression of lipid peroxidation. These effects collectively mitigated the occurrence of ferroptosis.

## Introduction

The skin, recognized as the largest organ of the human body, serves as the primary barrier against external environmental factors. Exposure to ionizing radiation inevitably leads to damage to the skin (Dąbrowska et al. 2018; Dörr et al. 2011; Lddins et al. 2022). RISI represents a significant complication in patients receiving radiotherapy and individuals exposed to nuclear accidents, leading to a prolonged wound-healing process relative to injuries caused by other factors (DiCarlo et al. 2020; Yang et al. 2020). The generation of ROS is identified as a primary etiological factor contributing to the development of RISI (Brand et al. 2017). Approximately 90% of patients receiving radiotherapy exhibit varying degrees of skin injury, with a gradual rise in occurrence (Yang et al. 2020; Brey et al. 2016). A significant majority, exceeding 60%, of individuals affected by the atomic bombings in Hiroshima and Nagasaki, as well as the Chernobyl nuclear disaster, experienced traumatic injuries, notably burns (Pellmar et al. 2017). The confluence of these injuries resulted in delayed wound healing.

Ferroptosis is a novel form of iron-dependently regulated cell death, distinguished by the intracellular accumulation of ROS (Stockwell et al. 2017; Tang et al. 2021). More specifically, polyunsaturated fatty acids (PUFAs) situated within mitochondria, endoplasmic reticulum, and lysosomes are susceptible to oxidation by lipoxygenase and ROS in the presence of Fe^2+^, leading to the formation of lipid hydroperoxides (L-OOH). This phenomenon induces lipid peroxidation and subsequent compromise of membrane integrity, ultimately resulting in cellular demise (Jiang et al. 2021; Conrad et al. 2018). Ferroptosis is known to be a significant factor in the pathogenesis of various diseases, including, carcinogenesis (Liang et al. 2019), infections (Chen et al. 2021), and ischemia-reperfusion injury (Fang et al. 2023). Several studies have demonstrated the pivotal involvement of ferroptosis in radiation-induced lung (Li et al. 2019) and intestinal (Wang et al. 2022) injury. Nevertheless, there is a notable scarcity of research examining the implications of ferroptosis in RISI.

Marveld3, a recently identified transmembrane protein featuring a quadruple transmembrane domain, is integral to the formation of tight junctions (Steed et al. 2009). Functioning as a signaling molecule, Marveld3 actively contributes to the regulation of cellular survival. Steed et al. have documented that Marveld3 downregulates the phosphorylation levels of c-Jun N-terminal kinase (JNK), thereby affecting essential cellular processes (Steed et al. 2014). Our previous research has demonstrated that the inhibition of Marveld3 is advantageous in suppressing the generation of radiation-induced intracellular ROS (Cao et al. 2021).

PRRX2 is a transcription factor that belongs to the paired family of homeobox proteins. It was initially identified in proliferating fetal fibroblasts and the developing dermal layer, where it plays a crucial role in fetal skin development and potentially influences cellular proliferation (Li et al. 2019a; Jiang et al. 2022). PRRX2 has also been implicated in the progression of glioblastoma through its regulation of ferroptosis (Jiang et al. 2022). However, to date, no studies have investigated the expression and functional implications of PRRX2 in RISI.

In this study, we investigated the correlation between Marveld3 and ferroptosis, discovering that the upregulation of PRRX2 serves as a stress response to irradiation. Subsequently, we observed that Marveld3 was implicated in ferroptosis of skin cells after irradiation by inhibiting lipid peroxidation. The potential underlying mechanism for this inhibition is suggested to be the overexpression of PRRX2.

## Materials and methods

### Reagents

Fer-1 (S7243) and Erastin (S7242) were purchased from Selleck Chemicals (Houston, TX, USA) and dissolved in dimethyl sulfoxide (DMSO).

### Cell culture and irradiation

The human keratinocyte cell line HaCaT and human skin fibroblast cell line WS1 were purchased from Deutsche Sammlung von Mikroorganismen und Zellkulturen GmbH (DSMZ; Germany) and American Type Culture Collection (ATCC, Gaithersburg, MD), respectively. The cells were cultured in dulbecco’s modified eagle medium (DMEM) supplemented with 10% fetal bovine serum (Biological Industries, Kibbutz Beit-Haemek, Israel) and antibiotics (100 units/ml penicillin G and 100 units/ml streptomycin sulfate; Beyotime, Jiangsu, China) at 37 °C in a 5% CO2 incubator. The cells received a 10 Gy dose with an X-ray linear accelerator (Rad Source Technologies Inc., Suwanee, GA) at a fixed dose rate of 1.15 Gy/min.

### Small interfering RNA (siRNA) or overexpression plasmid transfection

The sequences of Marveld3-specific siRNAs were found to be consistent with those previously described (5’-GAGAGGAGGUGGAAUAUUATT-3’). The efficacy of siRNA-induced knockdown of Marveld3 has been confirmed in prior studies (Cao et al. 2021). The siRNA sequence targeting the human PRRX2 cDNA (5’-GCCGCAGGAUGGUGAGUGUTT-3’) was designed and synthesized by GenePharma (Suzhou, China). The human PRRX2 overexpression plasmid was synthesized using the pcDNA3.1(+) vector by GenePharma (Suzhou, China). Lipofectamine 3000 transfection reagent (Thermo Fisher Scientific, Inc., MA, USA) was used for transient transfection of siRNA or plasmid, following the instructions provided by the manufacturer. The effects of transfection were assessed 72 h post-transfection.

### Cell viability assay

CCK-8 kit (#C0037, Beyotime, Jiangsu China) was used to measure cell viability. Cells in logarithmic growth were inoculated into 96-well plates at a density of 3 × 10^4^/well. After treatment, 10 ul of CCK-8 reagent (diluted in 100 µl of culture medium per well) was added to each well. The plates were then incubated for 1 h at 37 °C in a 5% CO_2_ incubator. The absorbance at a wavelength of 450 nm was measured using a microplate reader (Synergy2, BioTek, USA).

### MDA and Fe2+ detection

Lipid peroxidation serves as the primary mechanism underlying ferroptosis, with MDA emerging as a prominent end-product thereof (Stockwell et al. 2017). Intracellular levels of MDA were determined in accordance with the protocol provided by the manufacturer of the MDA assay kit (Cat #BC0025, Solarbio, Beijing, China). Additionally, the intracellular concentration of Fe^2+^ was quantified using the corresponding assay kit (Cat #BC5415) purchased from Solarbio (Beijing, China), following the instructions provided by the manufacturer.

### BODIPY detection

Lipid peroxidation was evaluated using a BODIPY 581/591 C11 kit (Cat#C10445, Thermo Fisher Scientific, Waltham, MA, USA). The BODIPY staining procedure was carried out in accordance with the manufacturer’s standard protocol. Confocal scanning laser microscopy (Olympus, Tokyo, Japan) was utilized to capture the images.

### Rat irradiation

The female Sprague-Dawley rats (6 weeks of age) were purchased from the Shanghai SLAC Laboratory Animal Co., Ltd. (Shanghai, China). Following anesthesia, a 3-cm-thick piece of lead was used to shield the rats and localize the radiation field (3 × 4 cm). The animals were exposed to a single 45 Gy dose to the treatment area at a dose rate of 750 cGy/min using a 6 MeV electron beam accelerator (Clinac 2100EX, Varian Medical Systems, Palo Alto, CA). The irradiated rat skin tissues were collected on days 1 and 7 post-irradiation, respectively, with an additional piece of skin obtained from a non-irradiated region of the same rat serving as a control.

### Immunohistochemistry

Immunohistochemistry was conducted to ascertain the expression of PRRX2 in skin tissues collected at various time points following irradiation of rats. Skin tissues were preserved in 10% neutral buffered formalin and subsequently embedded in paraffin. The paraffin-embedded skin tissue sections were subjected to immunohistochemical staining. Initially, the sections were immersed in a diluted primary antibody solution and incubated overnight at 4 °C. Subsequently, a secondary antibody (ZSGB-BIO Technology, Beijing, China) was applied for 30 min at room temperature. The sections were then developed using diaminobenzidine (DAB) staining and counterstained with hematoxylin for nuclear visualization. After staining, the sections were dehydrated and sealed with neutral balsam. The analysis of percentage and density of positively stained cells was conducted using Aipathwell, a software based on the artificial intelligence learning (Wuhan Servicebio Technology, Wuhan, Hubei, China). And the H-score method was used to evaluate the percentage of positively stained cells and staining intensity (H-score= (percentage of weak intensity × 1) + (percentage of moderate intensity × 2) + (percentage of strong intensity × 3) (Detre et al. 1995; Paschalis et al. 2019).

### Intracellular ROS production assay

The cells were subjected to the specified treatment and subsequently exposed to a fresh medium supplemented with 10 µM DCFH-DA (#S0033M, Beyotime, Jiangsu China) at a temperature of 37 °C for a duration of 30 min. Following this, the cells were rinsed thrice with PBS. Subsequently, the level of DCF fluorescence within the cells was quantified using a microplate reader (BioTekt Instruments, Winooski, VT) with an excitation wavelength of 488 nm and an emission wavelength of 525 nm.

### Western blot analysis

The cells underwent a series of procedures, including washing with ice-cold PBS, lysis in RIPA buffer, and centrifugation at 13,000 g for 10 min at 4 °C. The resulting supernatants were collected for Western blotting and subsequently loaded into a 10% SDS-PAGE gel. Electrophoresis of the samples was conducted for 2 h, followed by transfer onto PVDF membranes (Millipore, Bedford, MA). To prevent nonspecific binding, the membranes were blocked with 5% BSA in TBS-Tween-20 (0.1% TBST) at room temperature for 1 h. Subsequently, the membranes were incubated overnight at 4 °C with antibodies against Marveld3 (25667-1-AP, 1:1000), GPX4 (67763-1-Ig, 1:1000), DHODH (14877-1-AP, 1:1000), FSP-1(20886-1-AP, 1:1000), GCH1(20886-1-AP, 1:1000) (all from Proteintech). α-Tubulin (1:1,000 dilution; Beyotime, Jiangsu, China) was employed as a loading control. All antibodies were used at the concentrations indicated by the supplier. The antibodies were diluted to the appropriate concentration using Western primary antibody diluent (Beyotime, Jiangsu China). Subsequently, the membranes were incubated with the appropriate secondary antibody at a dilution of 1:1000, which was diluted in the blocking solution, for a duration of 1 h at room temperature. Following a wash with TBST, the blots were incubated in ECL (Beyotime Biotechnology) and the signals were detected using a FluorCheme M System (ProteinSimplet, San Jose, CA). Semiquantitative analysis was performed using ImageJ software (National Institutes of Health, Bethesda, MD).

### RNA extraction, reverse transcription and real-time PCR

Total cellular RNA was extracted using the RNA-Quick purification kit (Cat# RN001, ES Science, Shanghai, China). cDNA was synthesized from 2 µg of total RNA with a Reverse Transcription Kit (Cat#G490, abm, Vancouver, Canada). The expression of mRNAs within cDNA of PRRX2 was quantified by real-time PCR using the SYBR green kit (Cat#208054, Qiagen, Hilden, Germany), while GAPDH was employed as the internal reference gene for normalization. The real-time PCR was performed on the ViiA7 Real-Time PCR System (Applied Biosystems, Forster City, CA, USA). All steps were conducted according to the provided instructions. The primer sequences for PRRX2 consist of 5’-GCCGCAGGATGGTGAGTGTC-3’ for the forward primer and 5’-TCCGCCGCTGCTTCTTCTTC-3’ for the reverse primer.

### RNA-Sequencing experiments

The cells underwent the designated treatment, with three separate samples established for each group. TRIzol reagent, following the manufacturer’s protocols, was utilized to extract total RNA from the cells. Total RNA was quantified using a NanoDrop ND-2000 spectrophotometer (Thermo Scientifice, Wilmington, MA), and the integrity of the RNA was evaluated using an Agilent Bioanalyzer 2100 (Agilent Technologies, Santa Clara, CA). RNA sequencing, GO analysis and KEGG pathway analysis were performed by the OE Biotech Company (Shanghai, China).

### Statistical analysis

The data were presented as the mean ± standard deviation (SD). Student’s t-test was performed using GraphPad Prism version 8.0 (San Diego, CA). Statistically significant difference was considered when *P* < 0.05.

## Results

### Marveld3 is involved in radiation-induced ferroptosis of skin cells

**Fig. 1:**
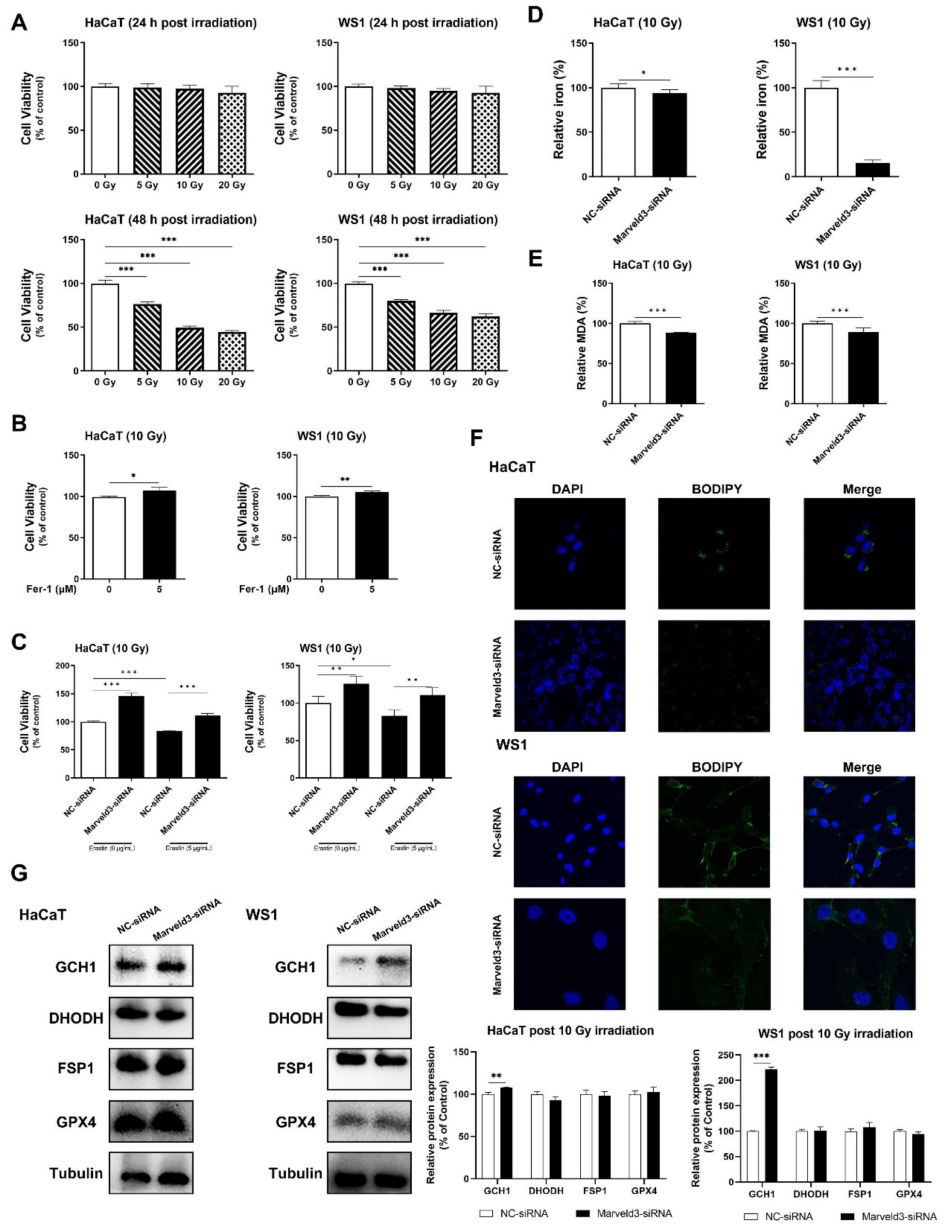
Marveld3 is involved in the radiation-induced ferroptosis of skin cells. **A**. The viability of HaCaT and WS1 cells were assessed following exposure to varying doses of irradiation at multiple time points by CCK-8 kit. **B**. HaCaT and WS1 cells were subjected to a 24-hour treatment of Fer-1 following exposure to 10 Gy X-ray irradiation, and the CCK-8 assay was used to detect cell viabilities. **C**. The cells were treated with erastin for 24 h after 10 Gy X-ray irradiation, and reversal of erastin’s effect on skin cell viability after irradiation was observed upon knockdown of Marveld3 using specific-siRNA. **D&E**. The contents of ferric ions and MDA were significantly reduced in the Marveld3 knockdown group compared to the group transfected with a negative control siRNA, detected with corresponding kits. **F**. Confocal images of C11-BODIPY stained HaCaT and WS1 cells showed lipid peroxidation status. **G**. Western blot assay was used to analyze the expression of the crucial molecules involved in the signaling pathways associated with the defense against ferroptosis after X-ray irradiation. Student’s t tests were used to evaluate differences between groups; *P < 0.05, **P < 0.01, and ***P < 0.001 compared to the control group

Initially, cell viability was assessed following exposure to varying doses of irradiation at different time points (Fig. 1A). A dose of 10 Gy was subsequently chosen for further experiments, with pertinent indicators assessed 48 h post-irradiation. Initially, irradiated skin cells were treated with Fer-1, a specific inhibitor of ferroptosis (Miotto et al. 2020). This treatment was observed to enhance cell viability following radiation exposure, thereby substantiating the occurrence of radiation-induced ferroptosis (Fig. 1B). Consequently, we investigated the potential influence of Marveld3 expression on the process of ferroptosis in skin cells. Erastin, a well-established agonist of ferroptosis (Zhao et al. 2020), exhibited additional suppressive effects on the viability of irradiated skin cells. However, our findings indicated that the inhibitory impact of erastin on cellular viability could be counteracted by attenuating Marveld3 expression (Fig. 1C). Additionally, we examined the Fe^2+^ levels in irradiated skin cells with varying levels of Marveld3 expression. It was observed that the Fe^2+^ content in Marveld3 knockdown cells was reduced following irradiation (Fig. 1D). We subsequently quantified the MDA content and observed a significant decrease in MDA levels in cells subjected to Marveld3 knockdown (Fig. 1E). Lipid peroxidation was evaluated using the BODIPY (581/591) C11 probe, which fluoresces green upon interaction with peroxidized lipids. The findings indicated a significant reduction in lipid peroxidation in cells with Marveld3 knockdown (Fig. 1F). Furthermore, we detected the impact of Marveld3 knockdown on the crucial molecules involved in signaling pathways that mediate ferroptosis defense. Among these, GCH1 showed the most pronounced upregulation (Fig. 1G).

### Differentially expressed genes in irradiated skin cells following the suppression of Marveld3

**Fig. 2:**
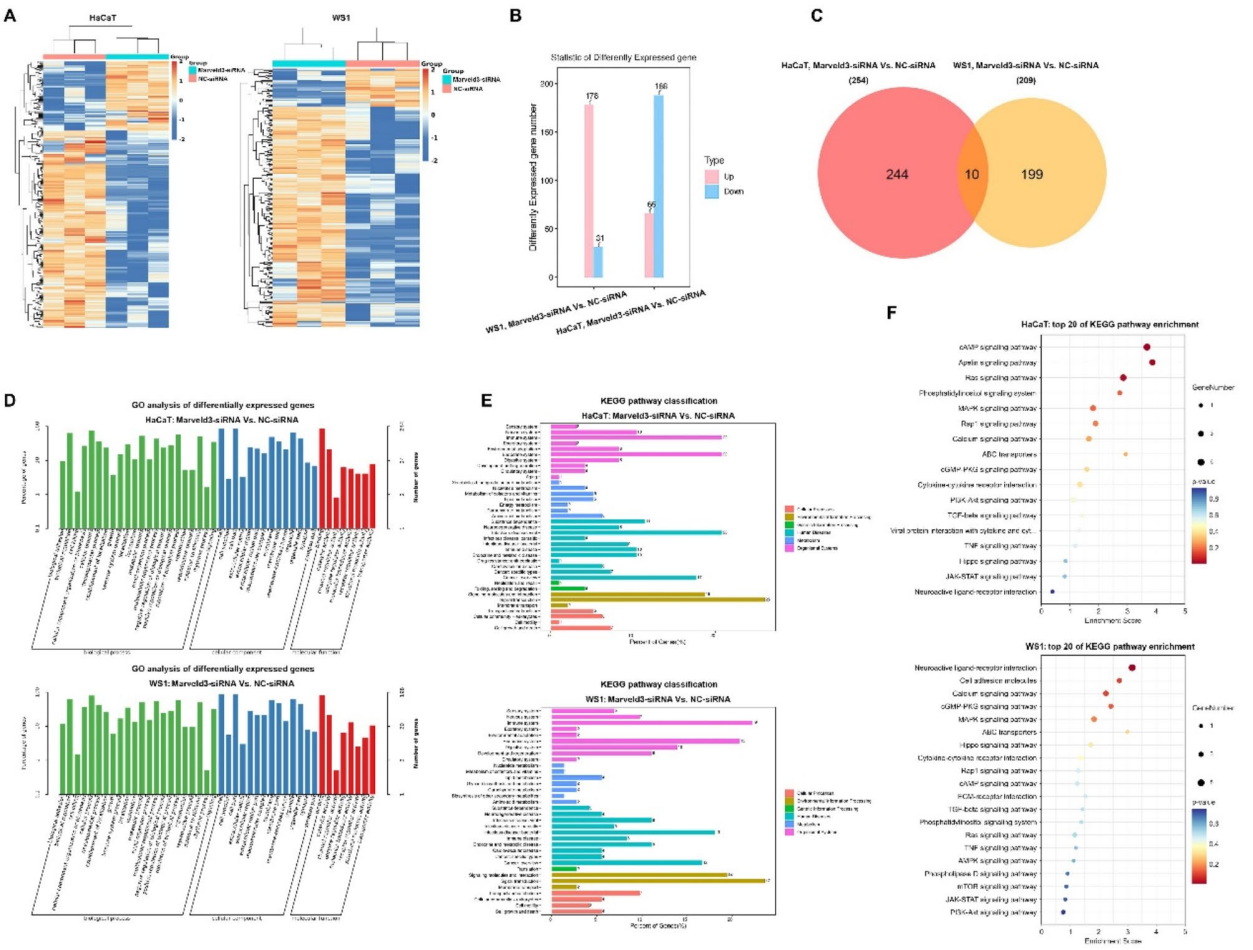
mRNA expression profiling of skin cells samples subjected to 10 Gy X-ray irradiation. HaCaT and WS1 cells were transfected with Marveld3-targeting siRNA or negative control siRNA, followed by exposure to 10 Gy X-ray. **A**. The heatmap of RNA-seq data illustrates the differentially expressed genes, with red indicating upregulated genes and blue signifying downregulated genes. **B&C**. The number of differentially expressed genes between the treatment and control groups in both HaCaT and WS1 cells is shown. **D**. GO category enrichment of the differentially expressed mRNAs in irradiated skin cells following Marveld3-knockdown. **E&F**. KEGG pathway enrichment of the differentially expressed mRNAs from irradiated skin cells in response to Marveld3-knockdown

To investigate the potential mechanisms underlying the influence of Marveld3 suppression on radioprotective effects, we performed an analysis of gene expression in irradiated HaCaT and WS1 cells with varying levels of Marveld3 expression using RNA-sequencing. The heatmap illustrated the distribution of differentially expressed genes in skin cells with varying levels of Marveld3 expression, with upregulated genes represented in red and downregulated genes in blue (Fig. 2A). In HaCaT cells, a total of 254 genes exhibited statistically significant alterations in their expression levels when comparing Marveld3 knockdown cells to the control group, with a minimum fold change of two (*P* < 0.05). Of these, 66 genes were upregulated and 188 were downregulated. Similarly, in WS1 cells, 178 genes exhibited upregulation, whereas 31 genes showed downregulation (Fig. 2B). Tables 1, 2, 3 and 4 demonstrated the expression levels of the top 20 up-regulated and top 20 down-regulated mRNAs in the Marveld3-knockdown group, in comparison to the control group, within the HaCaT and WS1 cells, respectively. Moreover, a total of ten genes exhibited statistically significant differences in expression in both cells. Notably, alterations in the expression of SLC22A1, ITGB2, LIMS4, and CCDC110 demonstrated consistent trends in both HaCaT and WS1 cells (Fig. 2C; Table 5).

To further investigate the differentially expressed genes, an analysis of GO enrichment analysis was conducted, which identified a significant enrichment of 44 GO terms in both HaCaT and WS1 cells. Additionally, separate GO enrichment analyses for each cell type yielded highly similar outcomes. In both cell types, the predominant categories within the biological process and cellular component were “cellular process’’, ‘‘biological regulation’’, and ‘‘regulation of biological process’’ (Fig. 2D). Furthermore, KEGG pathway analysis revealed that the differentially expressed genes were predominantly enriched in the categories of ‘‘Signal transduction’’, “Immune system”, and “Endocrine system” (Fig. 2E). Notably, the MAPK signaling pathway was identified as one of the top five pathways significantly enriched with differentially expressed genes across both cell lines (Fig. 2F).

**Table 1:** Upregulated mRNAs by silencing Marveld3 in HaCaT cells after 10 gy X-ray irradiation

mRNA name	Fold change	*p*-value	q-value
C5orf46	28.67	0.018	0.17
TMCO2	26.29	0.009	0.12
KCNK9	24.73	0.022	0.20
NXPE2	24.57	0.012	0.14
HCST	23.09	0.043	0.28
PRRX2	22.59	0.006	0.09
BNIP5	22.33	0.024	0.21
PHACTR1	19.11	0.039	0.27
TP53TG5	15.66	0.022	0.20
KCTD8	14.58	0.022	0.20
SPDYE18	13.84	0.009	0.12
HES5	13.70	0.030	0.24
GABRP	12.71	0.038	0.27
LOC105372440	12.58	0.045	0.29
CITED1	12.52	0.012	0.14
FIBIN	11.63	0.044	0.29
H2AC16	10.44	0.022	0.21
TBC1D3D	9.50	0.002	0.05
GNRHR	9.27	0.029	0.23
SLC29A4	8.87	0.031	0.24

**Table 2:** Downregulated mRNAs by silencing Marveld3 in HaCaT cells after 10 gy X-ray irradiation

mRNA name	Fold change	*p*-value	q-value
YPEL4	0.027	0.002	0.05
FBXO2	0.027	0.002	0.04
CYP4F2	0.032	0.018	0.18
TMEM190	0.034	0.006	0.10
LGSN	0.034	0.001	0.03
MATN1	0.034	0.019	0.19
FAM178B	0.034	0.006	0.10
H2AC21	0.036	0.007	0.10
SCT	0.039	0.031	0.24
SHISAL2B	0.039	0.043	0.29
PIFO	0.041	0.017	0.17
ZNF214	0.041	0.017	0.18
KIF1A	0.041	0.034	0.25
EFHC2	0.041	0.011	0.13
PRDM7	0.041	0.038	0.27
HTR6	0.044	0.043	0.28
HOXB8	0.044	0.045	0.29
GGN	0.045	0.004	0.07
ESAM	0.047	0.029	0.24
DGCR6	0.047	0.018	0.18

**Table 3:** Upregulated mRNAs by silencing Marveld3 in WS1 cells after 10 gy X-ray irradiation

mRNA name	Fold change	*p*-value	q-value
UTS2R	29.05	0.013	0.12
CYP4F12	27.65	0.013	0.12
GALNTL5	27.46	0.009	0.09
TMPRSS11E	25.66	0.012	0.11
GPR62	25.58	0.013	0.12
CDKL4	25.52	0.014	0.12
KRT13	24.37	0.007	0.08
KRT14	24.33	3.83E-05	0.002
LECT2	23.89	0.019	0.15
CACNA1H	23.76	0.020	0.15
METTL24	23.76	0.020	0.15
LCE4A	21.97	0.022	0.16
TULP1	21.90	0.026	0.18
FBXO47	21.82	0.039	0.23
CD86	20.25	0.048	0.26
SLC2A2	20.19	0.032	0.20
NRXN3	20.11	0.036	0.22
KRT71	19.79	0.012	0.11
SSX5	18.78	0.010	0.10
GAL3ST1	15.90	0.026	0.18

**Table 4:** Downregulated mRNAs by silencing Marveld3 in WS1 cells after 10 gy X-ray irradiation

mRNA name	Fold change	*p*-value	q-value
REG4	0.029	0.004	0.05
LDLRAD4	0.040	0.024	0.17
KRTAP4-9	0.043	0.017	0.14
FITM1	0.043	0.004	0.05
MYLPF	0.046	0.039	0.23
VWA3A	0.046	0.028	0.19
KRTAP4-7	0.047	0.034	0.21
CHI3L2	0.049	0.009	0.09
CD180	0.056	0.050	0.26
H1-5	0.064	0.026	0.18
C17orf64	0.077	0.045	0.25
CCNP	0.078	0.039	0.23
PKD1L3	0.078	0.047	0.26
GPR141	0.144	0.047	0.25
IDO1	0.162	0.030	0.19
RAI2	0.163	0.018	0.14
SPATA31A6	0.176	0.047	0.25
EIF3CL	0.185	3.00E-08	4.52E-06
ZNF497	0.191	0.004	0.05
SGPP2	0.208	0.022	0.16

**Table 5:** The intersection of differentially expressed mRNAs between HaCaT and WS1

mRNA name	HaCaT					WS1		
	Fold change	q-value	Regulation		Fold change		q-value	Regulation
SLC22A1	2.24	0.24	Up		9.60		0.20	Up
ITGB2	2.23	0.25	Up		4.03		0.26	Up
LIMS4	2.06	0.17	Up		2.75		0.07	Up
ATP2A3	0.46	0.24	Down		2.79		0.01	Up
CCDC110	0.30	0.23	Down		0.29		0.20	Down
C10orf105	0.20	0.14	Down		5.88		0.10	Up
TMEM37	0.19	0.24	Down		2.03		0.16	Up
ZNF467	0.17	0.01	Down		2.01		0.02	Up
H2BS1	0.09	0.31	Down		12.22		0.25	Up
GIMAP4	0.06	0.30	Down		2.87		0.01	Up

### The upregulation of PRRX2 expression serves as a stress-responsive to irradiation

**Fig. 3:**
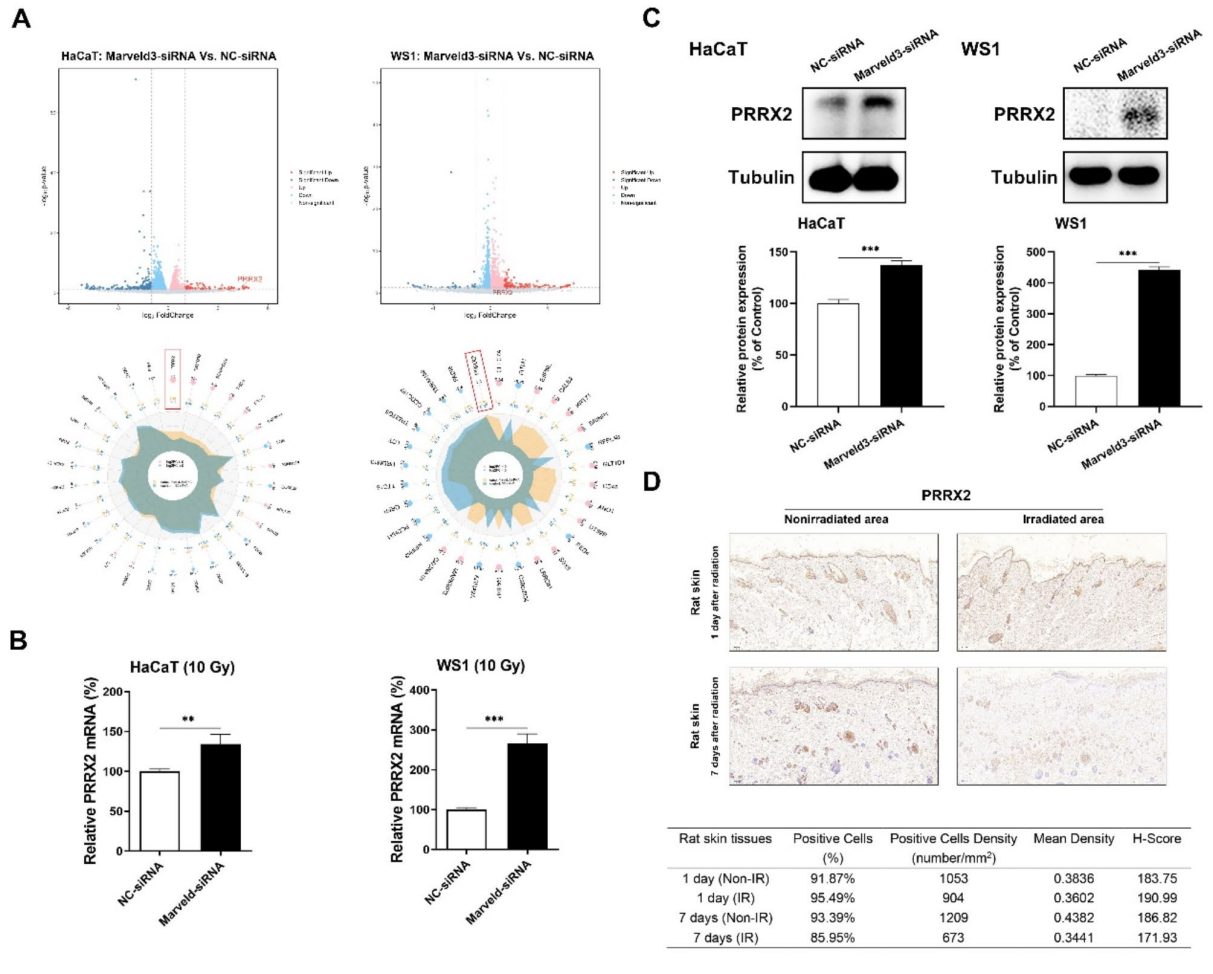
PRRX2 expression is upregulated in irradiated skin tissue, serving as a stress response. **A**. The volcano and radar plots of gene profiling between the Marveld3-targeting and negative control siRNA groups in HaCaT and WS1 cells. **B&C**. Confirmation of PRRX2 expression at both mRNA and protein levels was assessed using real-time PCR and Western blot techniques in Marveld3-knockdown skin cells. **D**. Immunohistochemical staining was used to detect the PRRX2 expression in both irradiated and nonirradiated rat skin tissues. Rat skin tissues were collected at days 1 and 7 following exposure to 45 Gy irradiation (*n* = 3). The AI software was utilized to conduct a quantitative analysis of PRRX2 positive staining in rat skin tissues, encompassing parameters such as the count of positive cells, density of positive cells, and H-Score, in both irradiated and nonirradiated samples

In HaCaT cells, previous research demonstrated a substantial elevation in PRRX2 expression, exhibiting an increase exceeding 20-fold compared to the control group. Additionally, PRRX2 exhibited the most statistically significant differences among the top 10 upregulated genes, with p-values less than 0.01 and q-values below 0.1, as presented in Table 1. The distribution of differentially expressed genes was illustrated using a volcano plot, where the red and blue points indicate statistically significant upregulated and downregulated mRNAs, respectively. Notably, PRRX2 expression was found to be upregulated in WS1 cells (Fig. 3A). In addition, it has been reported that PRRX2 is involved in ferroptosis (Li et al. 2019a), so that we chose PRRX2 for subsequent further investigations. The validity of the RNA-sequencing findings was corroborated through real-time PCR and Western blot analyses to evaluate mRNA and protein levels, respectively. Consistent results were observed in WS1 cells (Fig. 3B-C). We additionally observed the relative expression levels of PRRX2 in rat skin at various time points following exposure to a 45 Gy electron beam, alongside an analysis of immunohistochemical staining results in distinct regions of a single rat. One day post-irradiation, immunohistochemical analysis revealed that PRRX2-positive expression was elevated in irradiated skin tissues compared to normal skin tissues. Subsequently, the expression of PRRX2 in irradiated skin tissues progressively decreased, eventually falling below the levels observed in normal skin tissues (Fig. 3D). These results indicate that the upregulation of PRRX2 functions as a stress response to irradiation; however, its long-term sustainability appears to be limited.

### PRRX2 overexpression confers resistance to radiation-induced ferroptosis in skin cells

**Fig. 4:**
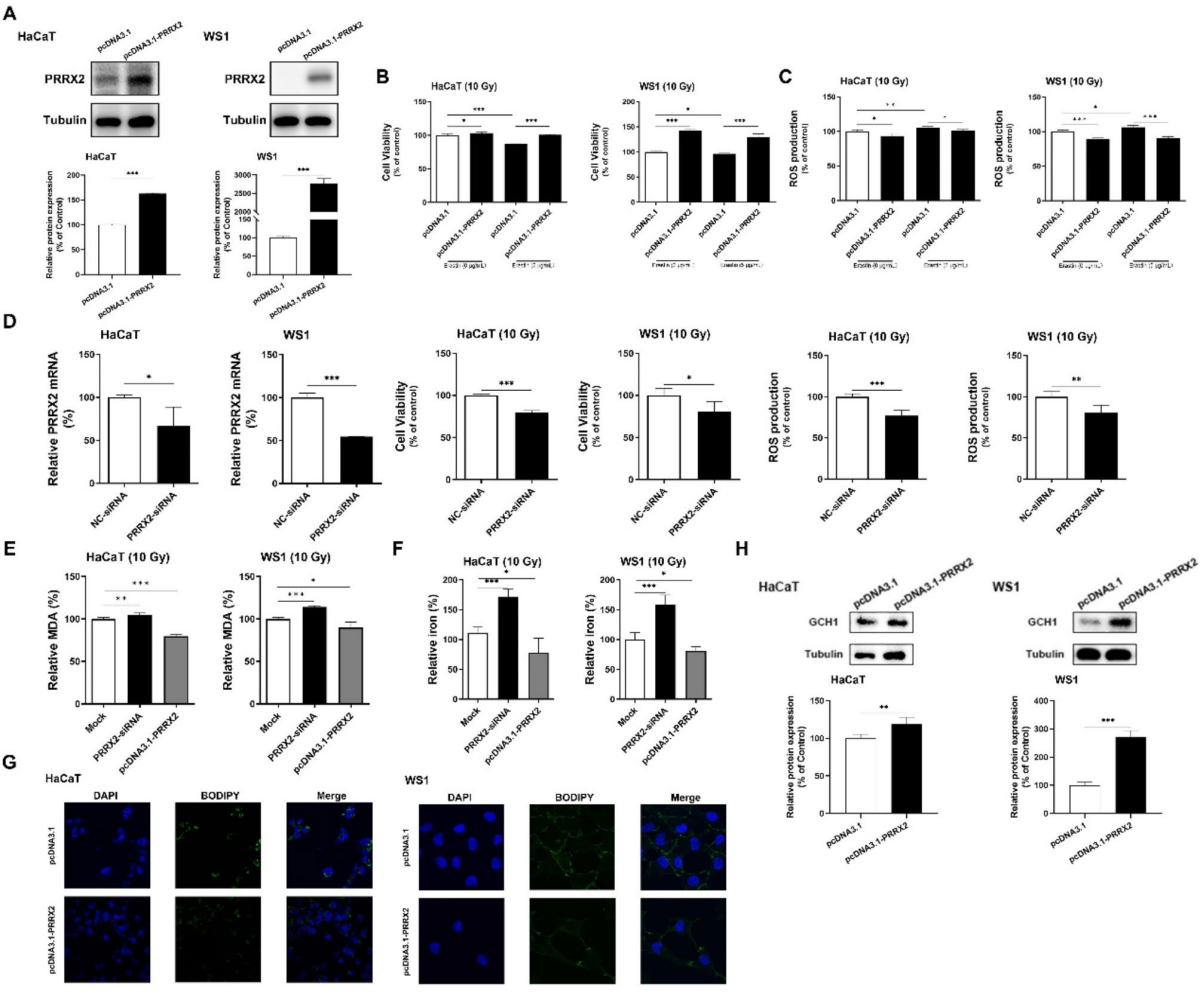
Overexpression of PRRX2 intervenes in the ferroptosis of skin cells induced by irradiation. **A**. The assessment of PRRX2 expression at the protein level was confirmed through Western blot techniques in HaCaT and WS1 cells, which had been transfected with a plasmid overexpressing PRRX2. **B**. Following 10 Gy X-ray irradiation, the cells were treated with erastin for 24 h. Upregulating PRRX2 expression reversed the effect of erastin on skin cell viability after irradiation by transfecting with a PRRX2-overexpressing plasmid. **C**. Determination of the ROS levels in HaCaT and WS1 cells transfected with PRRX2-overexpressing plasmid. The fluorescence intensity of DCF-DA reflects the concentration of ROS. The cellular level of DCF fluorescence was measured with a microplate reader at an excitation wavelength of 488 nm and an emission wavelength of 525 nm. **D**. The cells were transfected with PRRX2-siRNA, and cell viability as well as ROS generation were detected by specific kits. **E&F**. The levels of MDA and Fe^2+^ were assessed using specific kits, revealing a significant reduction in the PRRX2 overexpression group compared to the group transfected with a negative control plasmid. **G**. BODIPY staining was employed to assess the impact of PRRX2 overexpression on cellular lipid peroxidation levels. In HaCaT and WS1 cells. **H**. The impact of PRRX2 upregulation, induced by transfection with an overexpression plasmid, on GCH1. Student’s t tests were used to evaluate differences between groups; *P < 0.05, **P < 0.01, and ***P < 0.001 compared to the control group

The downregulation of Marveld3 exhibited a capacity to attenuate radiation-induced ferroptosis in skin cells and significantly upregulated the expression of PRRX2. Subsequently, we investigated the potential role of PRRX2 as a downstream target of Marveld3 in the context of ferroptosis. To this end, skin cells were transfected with an overexpression plasmid for human PRRX2 (pcDNA3.1-PRRX2) to induce elevated PRRX2 expression, while cells transfected with an empty vector plasmid served as controls (Fig. 4A). In comparison to the control group, skin cells overexpressing of PRRX2 demonstrated an elevation in cell viability upon exposure to 10 Gy X-ray irradiation (Fig. 4B). Moreover, the generation of ROS in PRRX2-overexpressing skin cells was comparatively diminished (Fig. 4C). In addition, PRRX2-specific siRNA was utilized to selectively downregulate PRRX2 expression in two distinct cell lines, resulting in the suppression of cell viability and elevation of intracellular ROS levels following irradiation (Fig. 4D). We quantified the levels of MDA and Fe^2+^ in cells exhibiting differential expression of PRRX2. Our findings demonstrated an inverse relationship between PRRX2 upregulation and the concentrations of MDA (Fig. 4E) and Fe^2+^ (Fig. 4F) in irradiated skin cells. To evaluate lipid peroxidation, BODIPY staining was utilized across HaCaT and WS1 cell lines with varying PRRX2 expression levels. Figure 4G showed that cells overexpressing PRRX2 exhibited a decreased fluorescence intensity. Moreover, our findings revealed the impact of PRRX2 overexpression on GCH1 expression, demonstrating an upregulation in both cell lines, with a notably more pronounced upregulation observed in WS1 cells (Fig. 4H).

### Differentially expressed genes in irradiated skin cells following the overexpression of PRRX2

**Fig. 5:**
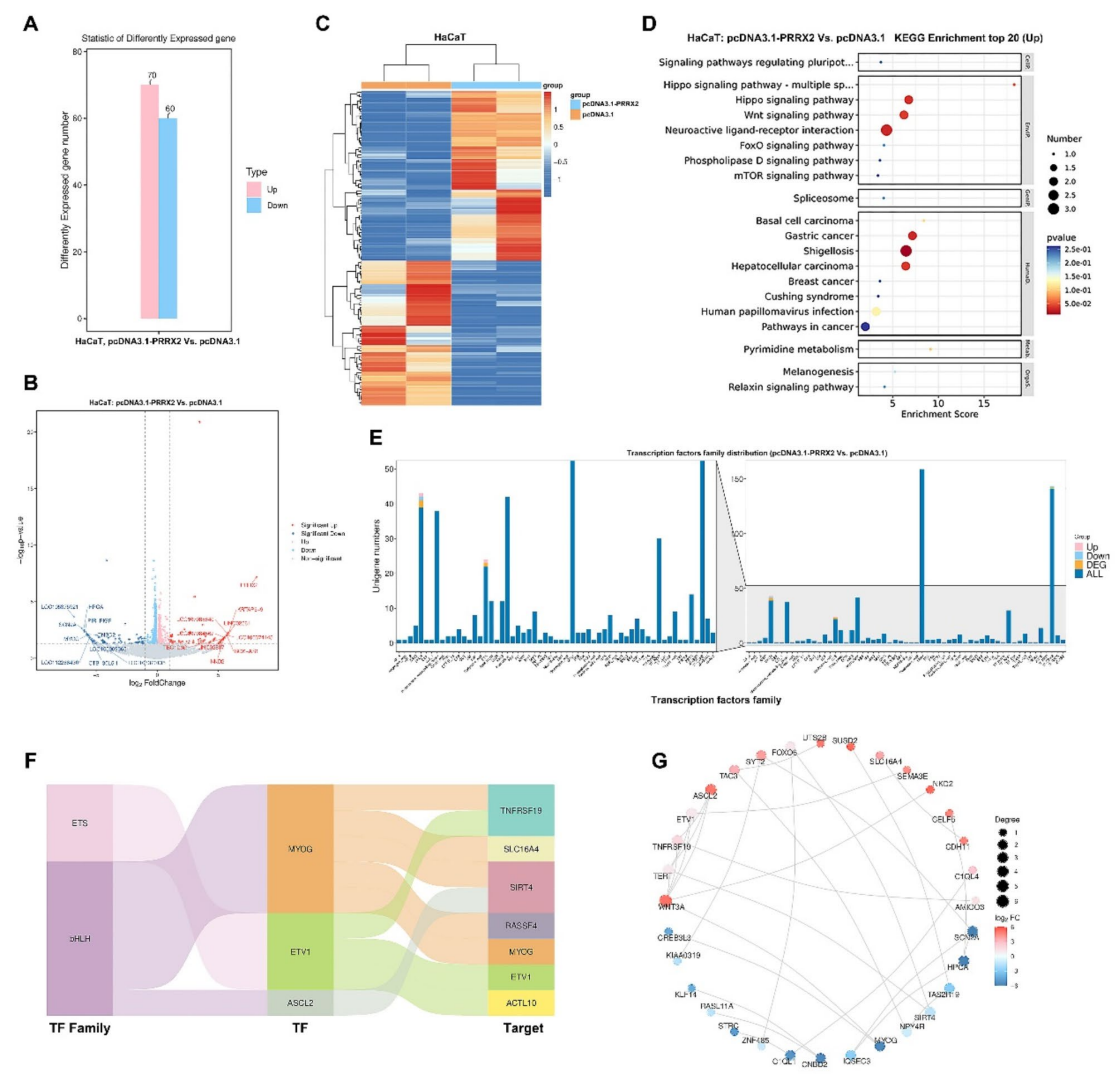
The impact of PRRX2 overexpression on mRNA expression profiles in irradiated HaCaT cells. **A**. The number of differentially expressed genes between the pcDNA3.1-PRRX2 group and pcDNA3.1 group in HaCaT cells is shown. **B**. The volcano plot of gene profiling between the pcDNA3.1-PRRX2 and control pcDNA3.1 groups in HaCaT cells. **C**. The distribution of differentially expressed genes in HaCaT cells following transfection with pcDNA3.1-PRRX2 and pcDNA3.1 was analyzed. Upregulated and downregulated differentially expressed genes are depicted by red and blue bars, respectively. **D**. KEGG pathway enrichment analysis was conducted on the significantly upregulated differential mRNA expression profiles derived from irradiated HaCaT cells. **E&F**. The alterations in transcription factor in differentially expressed genes resulting from PRRX2 overexpression, along with the prediction of pertinent target genes, were examined. **G**. Circular diagram of the top 30 interactions among differentially expressed genes consequent to PRRX2 overexpression

Given that PRRX2 exhibited upregulation in Marveld3-knockdown skin cells following irradiation, especially in HaCaT cells, we subsequently conducted an RNA sequencing analysis to investigate the underlying mechanisms of PRRX2 overexpression in HaCaT cells. The analysis revealed that a total of 130 genes exhibited statistically significant alterations in expression levels when comparing PRRX2-upregulated cells to the control group, with a minimum fold change of two (*P* < 0.05). Of these genes, 70 were upregulated, while 60 were downregulated (Fig. 5A-C). KEGG enrichment analysis revealed that the differentially expressed genes upregulated as a result of PRRX2 overexpression were predominantly associated with the Hippo and Wnt signaling pathways (Fig. 5D). Considering the function of PRRX2 as a transcription factor, we subsequently analyzed the changes in transcription factors among the differentially expressed genes resulting from PRRX2 overexpression. The results indicated that MYOG and ASCL2, both members of the bHLH transcription factor family, as well as ETV1 from the ETS transcription factor family, exhibited significant alterations in response to PRRX2 upregulation. Additionally, the target genes regulated by these differentially expressed transcription factors were subsequently predicted (Fig. 5E&F). We conducted a comprehensive analysis of the interactions among differentially expressed genes. Notably, Wnt3a, which exhibited a 35-fold upregulation compared to the control group, demonstrated interactions with six differentially expressed genes, representing the highest number of interactions observed in our study (Fig. 5G; Table 6). These findings strongly suggest that Wnt3a may play a pivotal role in radiation protection.

**Table 6:** Upregulated mRNAs by overexpressing PRRX2 in HaCaT cells after 10 gy X-ray irradiation

mRNA name	Fold change	*p*-value	q-value
PRRX2	275.85	7.06E-08	0.00018
LINC02561	62.42	0.003	0.37
KRTAP5-9	56.95	0.006	0.44
LOC107985840	54.22	0.007	0.49
BOK-AS1	51.74	0.010	0.56
LOC105374145	51.48	0.009	0.55
LOC107984567	45.90	0.015	0.68
NKD2	43.37	0.017	0.71
TBC1D3G	43.37	0.017	0.71
LINC02887	40.84	0.023	0.79
SUSD2	40.64	0.022	0.78
TTC34	38.00	0.028	0.84
MARCHF4	37.85	0.028	0.85
LOC107984341	37.80	0.029	0.86
LOC101929507	35.37	0.036	0.89
LOC105378482	35.32	0.035	0.89
WNT3A	35.01	0.041	0.92
ASCL2	32.73	0.049	0.96
UTS2B	32.73	0.049	0.96
RASSF4	32.68	0.047	0.95

## Discussion

Numerous instances of human exposure to high-dose radiation have been extensively documented, encompassing individuals affected by atomic bomb detonations in Hiroshima and Nagasaki, nuclear power plant catastrophes such as Chernobyl, as well as industrial and medical accidents (DiCarlo et al. 2020). In a considerable number of these exposures, the presence of skin injuries has been observed, exerting a substantial influence on the development of the injuries and the likelihood of survival following radiation exposure. Moreover, occurrences of radiation-induced skin complications have also been observed in routine clinical radiotherapy and radiation diagnostic imaging procedures (Lijima et al. 1982; Guskova et al. 1987; Zenda et al. 2016). While there are currently approved products available to mitigate radiation-induced hematopoietic complications, there remains a lack of approved treatment options for other injuries specifically related to radiation exposure. Notably, the skin has historically been the organ system most prominently affected in cases of human radiation exposure (DiCarlo et al. 2020). Marveld3, recognized as the third member of the occludin family within the Tight Junction-Associated Marvel Proteins (TAMPs), is integral to the regulation of epithelial paracellular permeability. It also plays a crucial role in modulating epithelial cell proliferation, migration, and survival, and is ubiquitously expressed across diverse tissues (Steed et al. 2009). In our previous study, we observed that Marveld3 significantly influenced the behavior of skin cells following irradiation. The inhibition of Marveld3 expression demonstrated a protective impact on cutaneous cells following radiation exposure, thus suggesting that Marveld3 is a potential target to ameliorate RISI (Cao et al. 2021).

Ferroptosis is a novel iron-dependent form of programmed cell death that, distinguished from other modes of cell death, exhibits distinct cellular morphology, mechanisms, and biochemical features (Stockwell et al. 2017; Tang et al. 2021). The biochemical attributes of ferroptosis encompass the intracellular accumulation of iron and ROS, the suppression of the cystine/glutamate transporter system, and the augmentation of NADPH oxidation, ultimately resulting in lipid peroxidation and the direct disruption of cellular membranes (Conrad et al. 2018; Liang et al. 2022). It was observed that the suppression of ROS production was apparent upon the targeted knockdown of Marveld3 using siRNA. Additionally, Marveld3, being a transmembrane protein, exerts an influence on the structure and function of the cellular membrane. Based on these findings, we hypothesized that Marveld3 plays a role in the occurrence of ferroptosis in skin cells post-irradiation. In order to validate this hypothesis, the current study was undertaken.

Firstly, it was observed that the application of Fer-1, a specific inhibitor of ferroptosis, resulted in an augmentation of skin cell viability post-irradiation, thereby indirectly validating the occurrence of radiation-induced ferroptosis in skin cells. Subsequently, our findings indicate that Marveld3 knockdown cells exhibited reduced levels of iron and MDA content. Furthermore, the suppression of Marveld3 effectively counteracted the impact of erastin, a well-known agonist of ferroptosis, on ferroptotic cell death. We conducted additional investigations into the expression of defense signaling molecules linked to ferroptosis (Liu et al. 2022; Fujii et al. 2023). Notably, only GCH1 expression demonstrated an increase, while the remaining molecules, including GPX4, FSP-1, and DHODH, did not exhibit any noticeable alterations.

Furthermore, to investigate the impact of Marveld3, we sought to identify the differentially expressed genes downstream. RNA-seq assays revealed that Marveld3-knockdown led to the downregulation of a majority of differentially expressed genes in HaCaT cells, while resulting in the upregulation of most differentially expressed genes in WS1 cells. Interestingly, only SLC22A1, ITGB2, LIMS4, and CCDC110 exhibited a consistent trend in both cell lines. Further research will be needed to examine the role of the aforementioned four genes in RISI. KEGG analysis revealed a significant enrichment of differentially expressed genes within the MAPK signaling pathway, attributable to the downregulation mediated by Marveld3. This phenomenon is likely due to Marveld3’s negative regulation of the JNK signaling pathway, achieved through modulation of JNK phosphorylation levels. Consequently, this regulatory mechanism impacts a series of cellular biological behaviors, including cell cycle progression, cell proliferation, and cell migration.

PRRX2, a member of the paired family of homeobox proteins that mediates epithelial to mesenchymal transition (EMT) and metastasis in breast and colon cancer (Juang et al. 2016; Chai et al. 2019). In the context of skin tissues, the expression of PRRX2 is specifically observed in actively dividing fetal fibroblasts and the maturing dermal layer, whereas its expression is attenuated in adult skin (Li et al. 2019a; Jiang et al. 2022). The upregulation of this gene during fetal wound healing, but not in adult wound healing, implies its potential involvement in regulating dermal regeneration in mammals and mitigating scar formation in response to injury. Our investigation revealed that PRRX2 was significantly upregulated in HaCaT keratinocytes upon Marveld3 downregulation, exhibiting the lowest p-value among the top 20 differentially expressed genes. Although PRRX2 was also upregulated in WS1 fibroblasts, the extent of upregulation was not as pronounced as in HaCaT. Consequently, we postulated that the overexpression of PRRX2 in adult skin cells confers advantageous effects on ameliorating RISI. Previous research has demonstrated that PRRX2 functions as a transcription factor and may exert transcriptional regulatory effects on GCH1, which plays a crucial role in mediating ferroptosis in glioblastoma. Jiang et al. further analyzed the potential transcription and binding sites of PRRX2 on the promoter region of GCH1 using the JASPAR database (Jiang et al. 2022). In light of these findings, we conducted transfection experiments using pcDNA3.1-PRRX2 in skin cells to observe cellular behaviors. The results obtained from this experiment indicated that PRRX2 is a potential downstream target of Marveld3, playing a crucial role in the regulation of ferroptosis. This process is predominantly mediated through the GCH1/BH4 signaling pathway, aligning with prior findings in glioblastoma research. Nevertheless, further investigation is required to elucidate the regulatory relationship between Marveld3 and PRRX2.

Previous research had found a connection between the oncogenic effects of PRRX2 and the activation of the Wnt/β-catenin signaling pathway (Chai et al. 2019). In our investigation, we also observed that the overexpression of PRRX2 resulted in the upregulation of Wnt3a significantly, a principal ligand of the canonical β-catenin signaling pathway. Furthermore, Wnt3a was found to interact with various differentially expressed genes induced by PRRX2 overexpression. The activation of the classical β-catenin signaling pathway by PRRX2-overexpression via Wnt3a may represent an additional mechanism through which it influences cell-related biological behaviors.

Our results showed that after downregulating Marveld3 in both cell lines, the differentially expressed genes were downregulated more in HaCaT and upregulated more in WS1. Additionally, the upregulation of PRRX2 was observed to be significantly higher in HaCaT cells (22-fold) compared to WS1 cells (approximately 2.5-fold), potentially attributed to the distinct tissue origins of the cell lines or the presence of p53 in WS1 cells but not in HaCaT cells. PRRX2 appears to exhibit a more pronounced upregulation in cells lacking functional p53.

## Conclusions

In conclusion, this study demonstrates the participation of Marveld3 in the process of radiation-induced ferroptosis in skin cells. The inhibition of Marveld3 led to the upregulation of PRRX2, which subsequently decreased the levels of ROS and Fe^2+^, inhibited lipid peroxidation, and thereby mitigated the occurrence of ferroptosis.

## Data Availability

No datasets were generated or analysed during the current study.
